# Measuring Neuromuscular Junction Functionality in the SOD1^G93A^ Animal Model of Amyotrophic Lateral Sclerosis

**DOI:** 10.1007/s10439-015-1259-x

**Published:** 2015-01-29

**Authors:** Emanuele Rizzuto, Simona Pisu, Antonio Musarò, Zaccaria Del Prete

**Affiliations:** 1 Department of Mechanical and Aerospace Engineering, Sapienza University of Rome, 00184 Rome, Italy; 2 Institute Pasteur Cenci-Bolognetti, DAHFMO-Unit of Histology and Medical Embryology, IIM, Sapienza University of Rome, 00161 Rome, Italy; 3 Center for Life Nano Science@Sapienza, Istituto Italiano di Tecnologia, 00161 Rome, Italy

**Keywords:** Muscle functional measurements, Nerve stimulation vs. direct stimulation, Neurotransmission failure, Intratetanic fatigue, ALS

## Abstract

Amyotrophic lateral sclerosis (ALS) is a fatal neurodegenerative disease that leads to motor neuron degeneration, alteration in neuromuscular junctions (NMJs), muscle atrophy, and paralysis. To investigate the NMJ functionality in ALS we tested, *in vitro*, two innervated muscle types excised from SOD1^G93A^ transgenic mice at the end-stage of the disease: the Soleus, a postural muscle almost completely paralyzed at that stage, and the diaphragm, which, on the contrary, is functional until death. To this aim we employed an experimental protocol that combined two types of electrical stimulation: the direct stimulation and the stimulation through the nerve. The technique we applied allowed us to determine the relevance of NMJ functionality separately from muscle contractile properties in SOD1^G93A^ animal model. Functional measurements revealed that the muscle contractility of transgenic diaphragms is almost unaltered in comparison to control muscles, while transgenic Soleus muscles were severely compromised. In contrast, when stimulated *via* the nerve, both transgenic muscle types showed a strong decrease of the contraction force, a slowing down of the kinetic parameters, as well as alterations in the *neurotransmission failure* parameter. All together, these results confirm a severely impaired functionality in the SOD1^G93A^ neuromuscular junctions.

## Introduction

Amyotrophic lateral sclerosis (ALS) is a disease that leads to motor neuron degeneration, muscle atrophy and paralysis.^23^,^43^ Although different pathogenic mechanisms have been proposed to account for ALS,^40^ several questions are still open and ALS remains an invariably fatal disease. The heterogeneity of the disease and failures in developing satisfactory therapeutic protocols reinforce the view that ALS is a multi-factorial and multi-systemic pathology.^34^,^47^ Recent studies on ALS animal models showed that the physiological connection between muscle and nerve is severely impaired,^16^,^34^ suggesting that the neuromuscular junction (NMJ) might be a direct target of the SOD1-mediated toxicity.^10^,^13^,^19^,^35^ Measuring NMJ functionality in ALS models, along with morphological and biochemical evaluations, should provide new insights on the physiopathologic interplay between muscle and nerve, and on the progression of the disease. Although the SOD1^G93A^ mouse is one of the most studied animal models for ALS,^21^ its skeletal muscle contractile properties have been poorly investigated and the NMJ functionality not elucidated. Previous studies on SOD1^G93A^ mice reported a deficit of the extensor digitorum longus (EDL) in generating maximum force after the onset of the pathology^12^,^24^ and a trend toward lower values of maximum specific force in diaphragm strips,^24^ when compared to wild type littermates. Those studies did not determine whether the alterations were due to changes either in muscle or in neuromuscular transmission.

Here we studied two different muscle-nerve preparations at the end-stage of the disease: the Soleus with its sciatic nerve, and the diaphragm with its phrenic nerve. The Soleus is a postural muscle composed of about 60% of fast fibers and 40% of slow fibers^5^ and is found to be almost completely paralyzed at the disease end-stage. On the other hand, the diaphragm is functional until death, usually caused by diaphragmatic failure and hypoventilation in both animal models and human patients.^17^

NMJ functionality can be measured, in isolated *in vitro* muscle-nerve preparations, by comparing the contractile response elicited by nerve electrical stimulation with the response of the same muscle evoked by direct stimulation of the membrane.^2^,^18^ Since this latter stimulation bypasses the neurotransmission signalling, any differences in the two contractile responses can be attributed to changes in the NMJ. Although this approach has been used in rats,^33^,^37^,^45^ there have been only a few studies in which it has been used in mice. Personius and Sawyer measured the diaphragm NMJ functionality in adult *mdx* mice,^36^ while Lee *et al*.^26^ and Ling *et al*.^27^ measured NMJ properties in the Soleus and in the EDL of an animal model of spinal muscle atrophy (SMA).

Thus, the main goal of this work was to evaluate the potential alterations of neuromuscular junction and muscle contraction in SOD1^G93A^ vs. wild type mice, analyzing the functional properties of two different muscle types. To accomplish this, we chose to measure the contractile properties of isometric twitches, the force-frequency relationship and the fatigue behavior.

## Materials and methods

### Experimental procedure

All experiments were conducted in accordance with the Italian Law which governs the use of animals in experimentation, and the procedure was approved by the local ethics review board. Both male and female wild-type C57BL/6 (WT) and transgenic SOD1^G93A^ mice were used in this study. The mice were sacrificed at 130–140 days of age. This age was chosen because it is close to the age at which SOD1^G93A^ mice normally die spontaneously. They were killed by cervical dislocation to minimize suffering. Immediately after sacrificing the mice, muscles were excised for testing. One muscle specimen was tested from each animal. The surgical excision of muscle-nerve samples was carried out in a dissecting bath containing a Bicarbonate—buffered storage solution^11^ continuously gassed with a mixture of 95% O_2_ and 5% CO_2_ at pH 7.4 and at room temperature.

The diaphragm: the muscle was isolated and the phrenic nerve was separated from the surrounding tissue for approximately 5 mm. The muscle and nerve were cut and removed. We then cut a strip of the excised muscle, for testing. The strip was cut parallel to the muscle fibers, with ribs at one end and central tendon at the other end, and was approximately 2–3 mm wide.

The Soleus: right and left Soleus muscles were removed along with 5 mm of their sciatic innervation. The Achilles’ tendon was cut as close as possible to the calcaneus and the entire triceps surae was removed. It was immediately put in the physiological bath and pinned to a silicon substrate. The proximal tendon of the Soleus was then gently pulled to separate the muscle from the surrounding tissues. Once the muscle was completely exposed, and the sciatic innervation separated, the distal tendon was cut. Both muscles were excised in order to increase the likelihood that at least one specimen would not have been damaged by the surgical procedure, but only one Soleus was tested for each animal.

Experiments were conducted using the following number of muscles: 11 WT and 8 SOD1^G93A^ Soleus, and 8 WT and 8 SOD1^G93A^ diaphragm strips. The muscle to be tested was vertically mounted in an oxygenated and temperature controlled chamber (30 °C for Soleus^3^,^11^ and 27 °C for diaphragm^36^) containing the same solution used in the dissecting bath. One end of the muscle was linked to a fixed clamp while the other end was connected to the lever-arm of a 300B actuator/transducer (Aurora Scientific Inc., ON, Canada). Each muscle was set up so it could be activated either by stimulating its nerve, or by direct stimulation. For nerve-evoked muscle activation, the nerve was sucked into a suction electrode (A-M Systems Inc., Sequim, WA) and supramaximal pulses were delivered to the nerve by a 701B stimulator (Aurora Scientific Inc., ON, Canada). For direct activation, two platinum electrodes were located 2 mm from each side of the muscle, while electrical stimuli were supramaximal current pulses of 300 mA, generated by a 701C stimulator (Aurora Scientific Inc., ON, Canada).^11^ A personal computer equipped with a NI PCIe-6353 acquisition board (National Instruments, Austin, TX) and programmed in LabVIEW 2012 (National Instruments, Austin, TX) synchronized the signals and the data acquisition for the entire length of the experiment. The custom-made software allowed for choosing all the stimulating parameters: the stimulating electrodes to use (nerve vs. direct), the pulse width, the stimulation frequency, the pulse train duration and the rest period between two consecutive pulse trains. The software was designed to guarantee high flexibility, accuracy, and repeatability of the stimulations. Synchronization between the signals delivered to the muscles and the acquired signals was evaluated with a TDS2014 digital oscilloscope (Tektronix Inc, Beaverton, OR). Results highlighted a maximum value of phase lag less than 10 *µ*s, which resulted negligible for the acquisition frequencies used in this study.

### Experimental protocol

To extensively investigate the neuromuscular junction functionality of Soleus-sciatic and diaphragm-phrenic nerve preparations, we established a continuous experimental protocol to measure several fundamental parameters of both muscle contractility and junction functionality.

Initial control experiments: we wished to demonstrate that (a) direct stimuli did not activate the muscle by stimulating intra-muscular branches of the nerve and (b) that stimuli applied to the nerve through the suction electrode did not activate the muscle artifactually, by spread of current in the bath. Therefore, in several control experiments utilizing both diaphragm and Soleus muscles, we measured muscle contractile response before and after adding d-tubocurarine (25 *µ*M) to the physiological bath.^30^ We found that d-tubocurarine completely silenced the specimen response to nerve stimulation, and did not significantly alter the muscle response to direct stimulation (data not shown). The above observations verified that the contraction caused by direct muscle stimulation was not mediated by the nerve stimulation pathway. In addition, we also wished to verify that the electric pulses delivered through the suction electrode did not spread in the solution, generating a current field strong enough to directly excite the muscle.^7^ Therefore, at the beginning of each experiment, we applied a few single pulses through the suction electrode before pulling the nerve in. We observed that supramaximal current pulses in the range of 5–10 mA did not excite the muscle membrane but, once the nerve was pulled in, were strong enough to activate the nerve and elicit muscle contraction. These were the current values we used for all nerve stimulation experiments.

In another set of preliminary experiments we determined the optimal pulse width for nerve stimulations, for both sciatic and phrenic nerves. We measured the relationship between the width of single stimulation pulses and the specific force, in ten Soleus and eight diaphragm muscle-nerve preparations. Pulse widths varied between 0.2 and 2.2 ms. In diaphragm, specific twitch force was maximal when using a pulse width of 1.8 ms, a value very close to that used by Personius and Sawyer^36^ (2.0 ms). In Soleus, specific twitch force was maximal using a pulse width of 1.4 ms. Since this value is slightly higher than that used by Lee *et al*.^26^ (1.0 ms) for Soleus in young mice, we performed additional preliminary experiments. We subjected seven Soleus muscles to three pulse trains at the tetanic frequency of 80 Hz: the first train was delivered directly to measure the maximum force, and the other two were delivered through the nerve with a pulse width of 0.2 and 1.4 ms, respectively. Results showed that only when stimulating the preparation with 1.4 ms pulses all the specimens were able to generate their maximum force. Accordingly to all these tests, we used a pulse width of 1.8 ms for phrenic nerve stimulation and 1.4 ms for sciatic nerve stimulation. Direct stimulation of diaphragm and Soleus used pulses of 0.2^30^ and 0.1 ms, respectively.^4^,^11^

**Figure 1:**
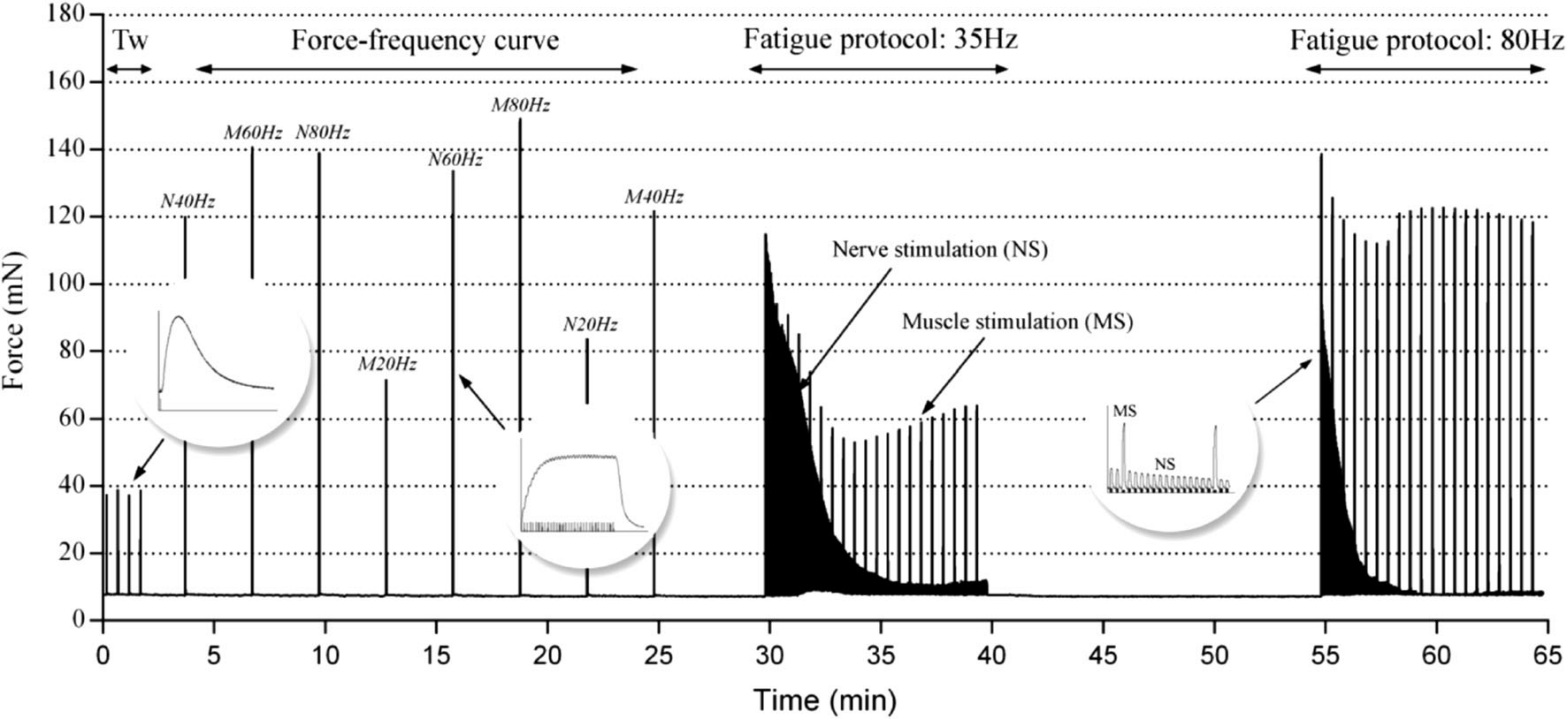
Example of a complete stimulation protocol showing the force values recorded from a wild-type Soleus. The muscle is initially stimulated with four single pulses (Tw), delivered alternatively one directly and one through the nerve. Subsequently, a series of eight pulse trains are delivered to the specimen at the indicated frequency, alternatively directly and through the nerve to measure the force-frequency curves. The protocol ends up with two fatigue paradigms: the first delivered at the firing frequency (35 Hz) and the second at tetanic frequency (80 Hz). Insets show stimulation pulses and force response curves on a magnified time scale

Once the optimal stimulation parameters were determined, we designed an experimental protocol to gather all the desired data in the shortest time. The protocol we ended up with consists of four parts: (1) a single pulse stimulation sequence, (2) an evaluation of the force-frequency relationship, (3) a fatigue paradigm at a physiologic firing frequency, and (4) a fatigue paradigm at the fused tetanic frequency. While the protocol structure is the same for the two muscle types, the stimulation parameters, namely the pulse width, the train duration and the rest periods, differ when the protocol refers to Soleus or diaphragm muscles. An example of the forces measured during the entire protocol for a wild-type Soleus is shown in Fig. 1. In the first part, the muscle was stimulated with four single pulses, one delivered directly and one through the nerve, repeated twice, with a rest period of 30 s between each. Time to peak (TTP), half relaxation time (½*RT*), maximum value of force derivative (*dF*/*dt*) and twitch force (*F*_tw_) were then measured from the twitch responses. After a rest period of 120 s, we stimulated the specimen with a series of pulse trains in the range between 20 Hz and the tetanic frequency, to compute the force-frequency curves for both nerve and direct stimulations. In particular, since the tetanic frequency is 80 Hz for Soleus^6^ and 100 Hz for diaphragm,^36^ we stimulated all the specimens using the following shuffled sequences: for Soleus muscles, N40 Hz (through the nerve), M60 Hz (direct on the membrane), N80, M20, N60, M80, N20, and M40 Hz; for diaphragm muscles, N60, M100, N40, M20, N80, M80, N20, M40, N100, and M60 Hz. The rest periods imposed after each stimulation were established with preliminary experiments. For each muscle, the *un*-*fatigued* maximum force was measured and taken as reference value. The rest periods were then determined as the minimum pause that allowed the muscle to recover its capability of generating maximum force. For Soleus muscles this rest time was equal to 3 min for all the stimulation frequencies, while for diaphragm muscles it was possible to reduce the rest periods accordingly to the stimulation frequency: 180 s rest time after tetanic stimulation, 150 s after the 80 Hz stimulation and only 120 s after all the other stimulations. After a rest time of 5 min, in the third and fourth parts of the protocol, the muscle was subjected to two fatigue paradigms in order to measure the *neurotransmission failure* (*NF*) at two different frequencies.^25^,^36^ The rest time between the two fatigue paradigms was considered appropriate when the force generated by the muscle at the beginning of the tetanic fatigue paradigm was at least 90% of the maximum force the same specimen generated during the force frequency measurements. We estimated an appropriate rest time of 15 min for both muscle types, as shown in Fig. 1 for a wild-type Soleus muscle.

Diaphragm stimulation: the parameters used during the first fatigue paradigm to measure the *neurotransmission failure* at the firing frequency were derived from Personius and Sawyer^36^: one 0.33 s pulse train at 35 Hz delivered directly, followed by fourteen 0.33 s pulse trains at 35 Hz delivered through the nerve, with a rest period of 0.67 s after each pulse train. This sequence was repeated 20 times for an overall stimulation period of 5 min.

Soleus stimulation: the protocol parameters were determined with preliminary experiments. In fact, because of Soleus fiber composition,^15^,^41^ longer pulse trains are required to stimulate the muscle at its maximum force. The resulting fatigue paradigm at firing frequency was as follows: one 0.8 s pulse train at 35 Hz delivered directly, followed by fourteen 0.8 s pulse trains at 35 Hz delivered through the nerve, with a rest period of 1.2 s after each pulse train. Just as for the diaphragm, this sequence was repeated 20 times, resulting in an overall fatigue paradigm time of 10 min.

The *neurotransmission failure* was then computed for both muscle types as $NF = \frac{F - MF}{1 - MF}\%$, where *F* is the force decrease measured after nerve stimulation and *MF* is the force decrease following muscle stimulation.^25^,^36^

For the second fatigue paradigm, the pulse train duration, the rest period and the number of repetitions were the same as in the previous one, but the stimulation frequency was now set to 80 Hz for the Soleus and to 100 Hz for the diaphragm. During this paradigm, we also measured the *intra*-*tetanic fatigue* (*IF*) as proposed by van Lunteren *et al*. for rat diaphragm.^45^ *IF* is given by the force at the end of the pulse train as percent of the maximum force generated during that same pulse train. As regards to the nerve stimulation, the intra-tetanic fatigue was computed on the first pulse train immediately after the direct stimulation. The entire length of the protocol was about 65 min for the Soleus and about 55 min for the diaphragm. Of note, in a previous paper we demonstrated that *ex vivo* skeletal muscle preparations kept in an appropriate bath, did not undergo any significant alterations over comparable time periods even if subjected to highly demanding stimulation protocols.^11^

For each experiment, the initial muscle length was adjusted to the length (*L*_0_) which produced the highest twitch force. At the end of the protocol, net muscle length and weight were measured using an analog calliper, with an accuracy of 0.05 mm, and a Pioneer precision scale (Ohaus, Parsippany, NJ), with an accuracy of 0.1 mg. Cross Sectional Area (CSA) for force normalization was estimated dividing the muscle mass by the product of $L_{\text{f}}$ and the density of mammalian skeletal muscle (1.06 mg/mm^3^). $L_{\text{f}}$ is the optimal fiber length and was obtained by multiplying $L_{0}$ for the *fiber length to the muscle length ratio* (0.71 for Soleus^6^ and 1 for diaphragm muscle^31^).

### Statistical Analysis

Statistical analyses were performed separately on Soleus and diaphragm data. Differences in the values of muscle weight, length and CSA were evaluated with Student’s *t* tests. Since in our analyses we studied a continuous outcome on the basis of categorical variables, we referred to Analysis of Variance to identify any significant differences between the means of tested groups. Differences in the values of TTP, ½*RT* and *dF*/*dt* were evaluated with 2-way ANOVAs using the factors *animal* *strain* and *stimulation type*. When the 2-way ANOVA indicated significant differences, we used multiple *t* tests to look for statistical differences. Similarly, differences in force-frequency curves and in the intra-tetanic fatigue were evaluated with a 3-way ANOVA using the factors *animal strain*, *stimulation type* and *frequency of stimulation* or *time*. When the 3-way ANOVA indicated a significant effect of the factors *animal strain* and *stimulation type*, we applied a 2-way ANOVA to identify significant differences between transgenic and control specimens stimulated directly, and between transgenic specimens stimulated directly and through the nerve. Differences in the neurotransmission failure were evaluated using a two-way ANOVA with reference to the fixed factors *stimulation type* and *time*. Statistical analyses was performed with GraphPad Prism 6.0 and SPSS 17.0 and differences were considered significant when *p* < 0.05.

## Results

Values of muscle weight, length and estimated CSA are reported in Table 1 for SOD1^G93A^ and WT Soleus. Transgenic muscles from 130 to 140 day-old mice displayed, on average, a 24% decrease in muscle weight (*p* < 0.001) and a 7% decrease in muscle length (*p* < 0.05), which resulted in a decrease of 18% of muscle CSA (*p* < 0.01), in comparison to wild type littermate.

**Table 1:** Anatomical measurements for WT and SOD1^G93A^ Soleus

Soleus	Weight (mg)	Length (mm)	CSA (mm^2^)
WT	7.77 ± 0.32	10.92 ± 0.18	0.94 ± 0.03
SOD1^G93A^	5.88 ± 0.33	10.18 ± 0.22	0.77 ± 0.04

Values are mean ± SEM

**Figure 2:**
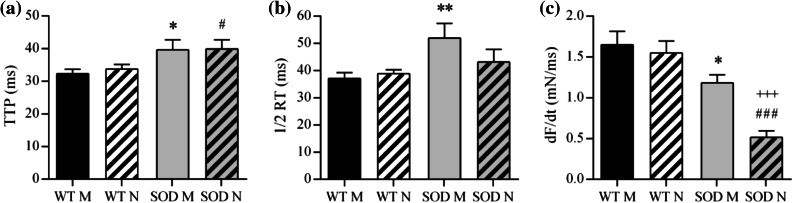
Twitch response properties of control (WT) and transgenic (SOD) Soleus muscles stimulated directly (M) and through the nerve (N). Shown are the mean ± SEM of the TTP (a), the ½RT (b) and the dF/dt (c). SOD is for SOD1^G93A^. * and **: *p* < 0.05 and *p* < 0.01 vs. WT M; ^#^ and ^###^: *p* < 0.05 and *p* < 0.001 vs. WT N; ^+++^: *p* < 0.001 vs. SOD M

**Figure 3:**
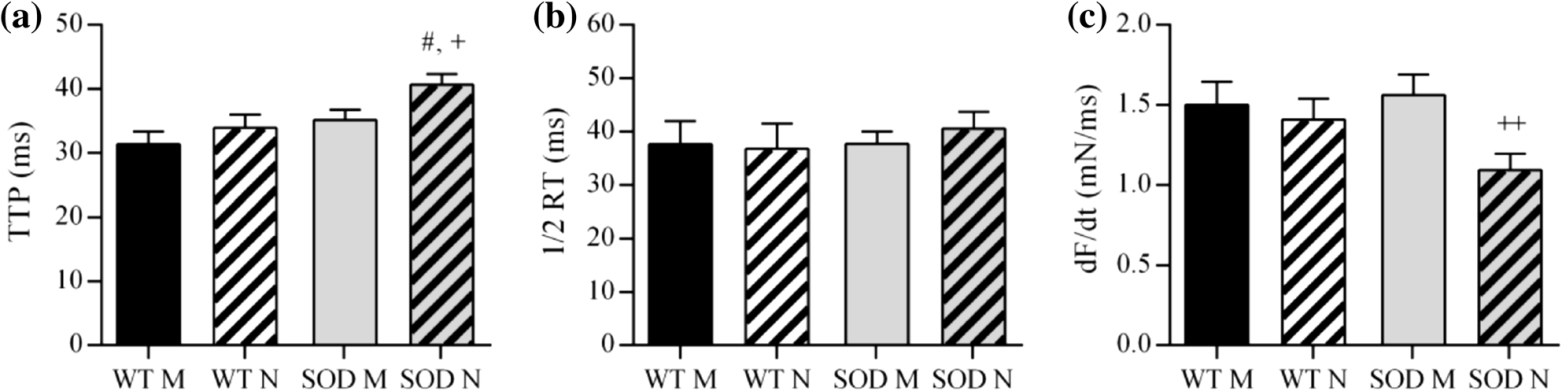
Twitch response properties of control (WT) and transgenic (SOD) diaphragm muscles stimulated directly (M) and through the nerve (N). Shown are the mean ± SEM of the TTP (a), the ½RT (b) and the dF/dt (c). SOD is for SOD1^G93A^. ^#^: *p* < 0.05 vs. WT N; ^+^ and ^++^: *p* < 0.05 and *p* < 0.01 vs. SOD M

Twitch response features of Soleus muscles stimulated directly and through the nerve in WT and SOD1^G93A^ animals are shown in Fig. 2. TTP (Fig. 2a) and ½*RT* (Fig. 2b) were significantly greater in SOD1^G93A^ muscles, indicating that the isometric twitch was slower to rise and slower to decay than in WT. SOD1^G93A^ muscles also had a lower rate of force generation, *dF*/*dt* (Fig. 2c), and *dF*/*dt* was even smaller with nerve stimulation than with direct stimulation. Twitch response features of WT and SOD1^G93A^ diaphragm muscles stimulated directly and through the nerve are shown in Fig. 3. Transgenic diaphragms showed significantly longer TTP (Fig. 3a) and lower *dF*/*dt* (Fig. 3c) only in nerve-evoked twitches.

**Figure 4:**
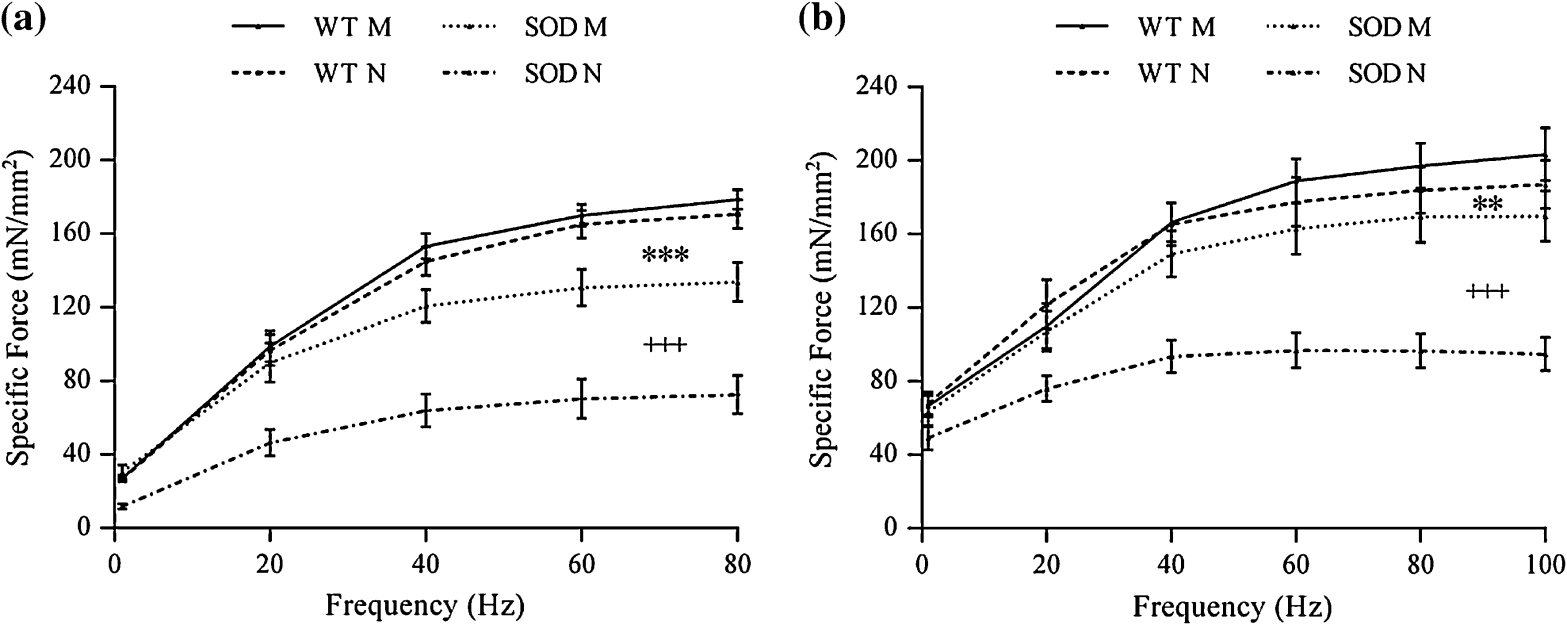
Relationship between specific force and frequency of stimulation from single pulse to tetanus, for Soleus (a) and diaphragm (b) specimens. M indicates direct membrane stimulation, N indicates nerve stimulation. For both muscle types, the SOD M curve was significantly different from the WT M and the SOD N curve was significantly lower than the SOD M. Values are mean ± SEM. SOD is for SOD1^G93A^. ** and ***: *p* < 0.01 and *p* < 0.001 vs. WT M; ^+++^: *p* < 0.001 vs. SOD M

Force-frequency curves, from single pulse to tetanic frequency, are shown in Fig. 4. Both Soleus (Fig. 4a) and diaphragm (Fig. 4b) SOD1^G93A^ muscles developed specific forces significantly lower than WT, regardless of stimulation frequency. Direct stimulation of SOD1^G93A^ Soleus muscles revealed a reduction, on average, of 25% (*p* < 0.001) in the maximum specific force when compared to WT. Interestingly, a further average decrease of 46% (*p* < 0.001) was found for SOD1^G93A^ Soleus maximum specific force measured with nerve stimulation, when compared to the response of the same transgenic muscle stimulated directly. Transgenic Soleus maximum specific force measured by nerve stimulation was then reduced by 57%, on average, when compared to WT (*p* < 0.001). A generally similar result was observed when testing diaphragm strips (Fig. 4b). Moreover, a significant difference (*p* < 0.01) emerged between the force-frequency curves measured for transgenic and WT muscles when stimulated directly. For SOD1^G93A^ muscles there was also a significant difference between the direct and the nerve stimulation curves (*p* < 0.001). Analysis of SOD1^G93A^ diaphragm maximum specific forces showed only a trend toward lower values when directly stimulated (*p* ~ 0.1), while a strong decrease of 44%, on average, was found when diaphragm was stimulated through the nerve (*p* < 0.001). Therefore, SOD1^G93A^ diaphragm maximum specific force measured through nerve stimulation resulted reduced, on average, of 50% when compared to WT diaphragm stimulated in the same way (*p* < 0.001).

**Figure 5:**
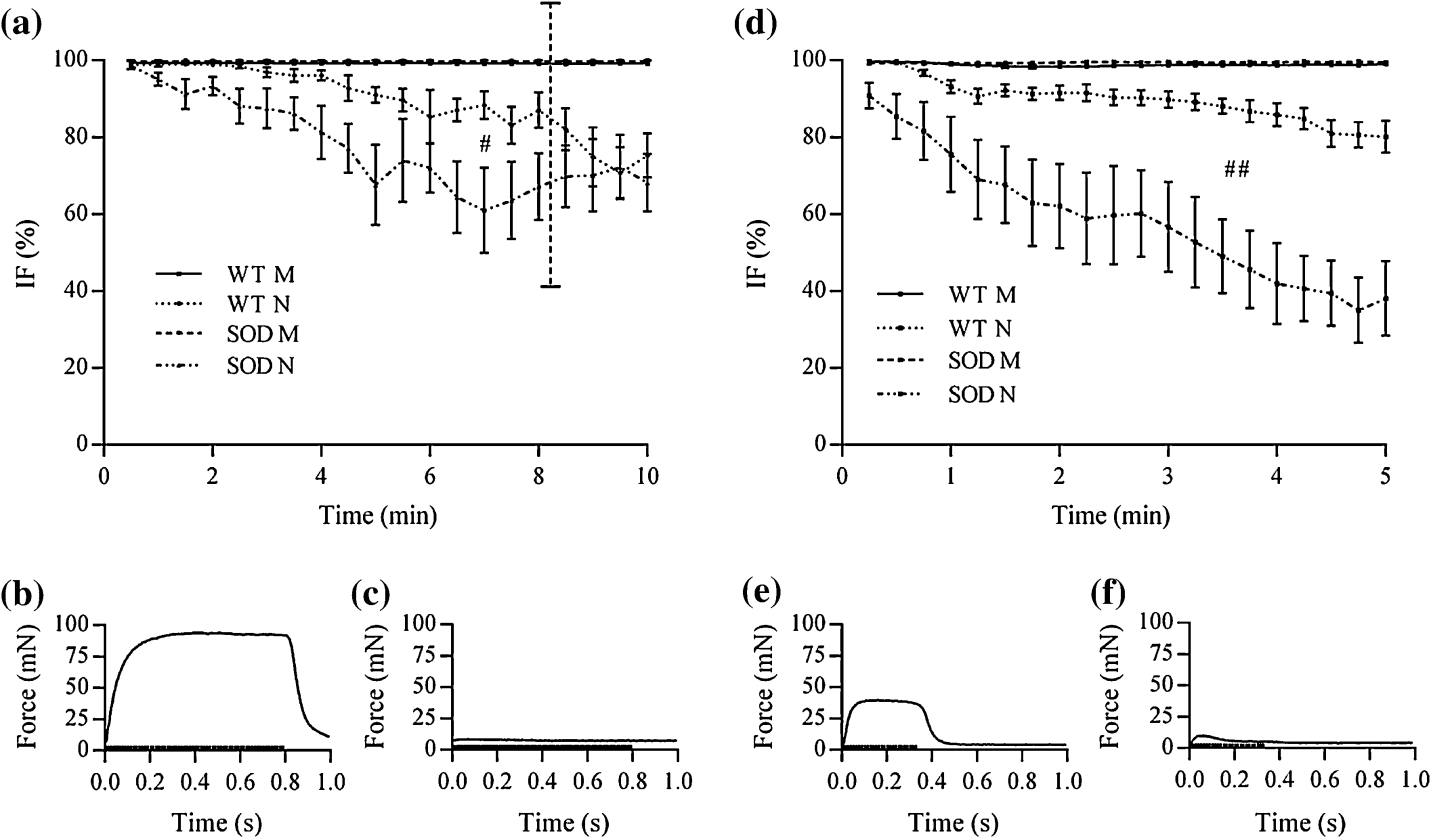
Analysis of *intratetanic fatigue* computed for Soleus (a) and diaphragm (d) muscles. Force values measured with nerve stimulation at the beginning of the protocol allows for a reliable *IF* measurement for both SOD1^G93A^ Soleus (b) and diaphragm (e). At the end of the protocol the force generated by transgenic Soleus drops down near zero (c) therefore, from this point on, the values of *IF* expresse noise. Thus, statistical analysis has been performed taking into account only the first 8 min of stimulation, as indicated by the dotted line in (a), were the contraction forces are higher than 5 mN. On the contrary, forces generated by diaphragm specimens allow for a good *IF* measurement until the end of the protocol (f). Values are mean ± SEM. SOD is for SOD1^G93A^. ^#^ and ^##^: *p* < 0.05 and *p* < 0.01 vs. WT N

Figure 5 shows the average values of *intratetanic fatigue* (*IF*) measured during the tetanic fatigue paradigm for Soleus (Fig. 5a) and diaphragm strips (Fig. 5d). For both muscle types, the 3-way ANOVA revealed that all the fixed factors, *animal strain*, *stimulation type* and *time*, significantly affected the *IF*. Neither Soleus nor diaphragm, in both WT and SOD1^G93A^ animals, exhibited any *intratetanic fatigue* when studied with direct stimulation. Therefore, the *IF* results described here below are only for nerve stimulation experiments. Regarding Soleus muscle, the 2-way ANOVA revealed that *IF* was significantly affected only by the fixed factor *time* (*p* < 0.001). A closer analysis of the force curves measured for each pulse train highlighted a limitation of the method when computing *intratetanic fatigue* at the end of the protocol. At the beginning of the 80 Hz fatigue paradigm the forces generated by transgenic muscles were always higher than 80 mN (see Fig. 5b) allowing a reliable measurement of the force decrease within the same pulse train. At the end of the paradigm, on the contrary, the muscles were exhausted and the contraction forces were near zero, especially for atrophic muscles like SOD1^G93A^ (see Fig. 5c): in this situation, the sensitivity of the method was reduced to zero and the *IF* values obtained with the calculation, from this point on, basically expressed noise. Therefore, the 2-way analysis of variance was performed taking into account only the first 8 min of stimulation of the 80 Hz fatigue paradigm, where the measured forces were always higher than 5 mN. Within this limitation, *IF* was significantly affected also by the fixed factor *animal* *strain* (*p* < 0.05) and was lower in the SOD1^G93A^ group. Statistical analysis of *IF* measured during the 100 Hz fatigue paradigm for diaphragm strips revealed that *IF* was significantly affected by the fixed factors *time* and *animal* *strain*, even taking into account the values measured until the end of the paradigm. In fact, WT and transgenic diaphragm specimens were able to generate remarkable forces both at the beginning (Fig. 5e) and at the end (Fig. 5f) of the fatigue paradigm. Finally, it should be noted that a very high variability was observed for *IF* in both transgenic Soleus and diaphragm specimens when stimulated through the nerve.

**Figure 6:**
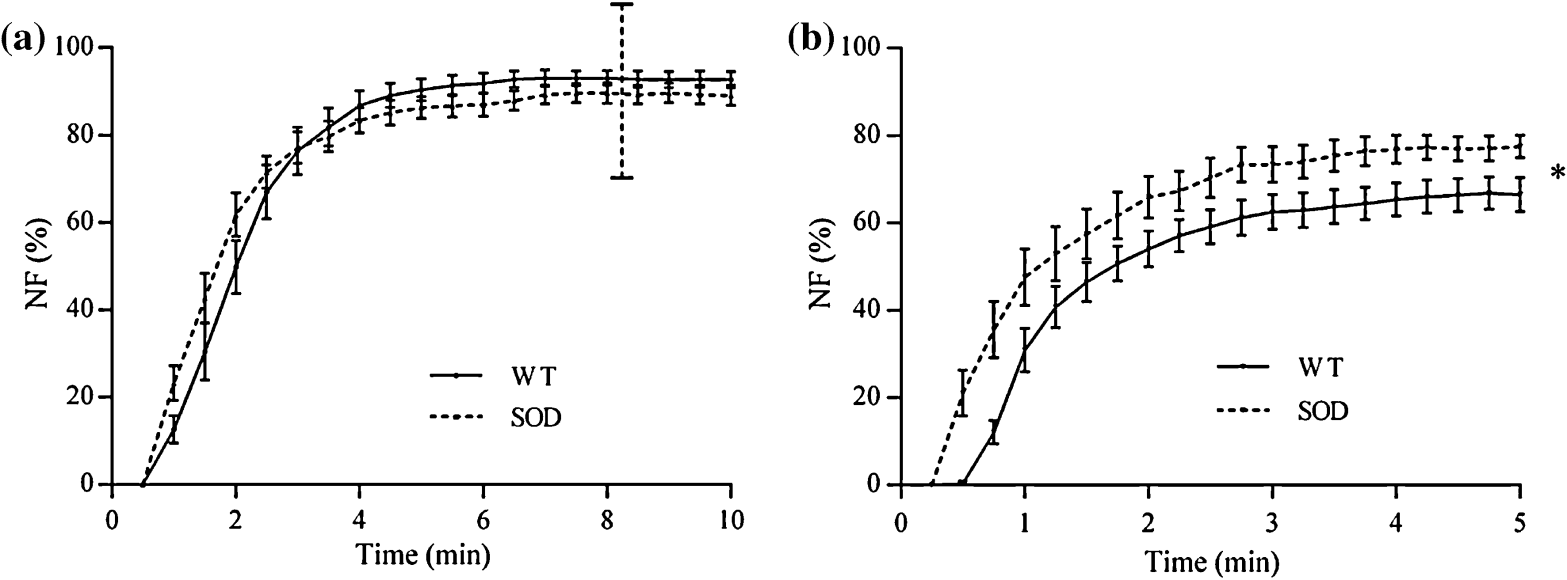
*Neurotransmission failure* computed at the tetanic frequency of 80 Hz for Soleus (a) and 100 Hz for diaphragm (b) specimens. *NF* values for Soleus muscle should be considered reliable only until the 8th min of stimulation, as indicated by the dotted vertical line (a), where the forces generated by the muscle specimens were higher than 5 mN. Values are mean ± SEM. SOD is for SOD1^G93A^. *: *p* < 0.05 vs. WT

Figure 6 shows the *neurotransmission failure* at tetanic frequency measured for Soleus (Fig. 6a) and diaphragm (Fig. 6b) specimens. Soleus *NF* was affected only by the fixed factor *time*, while diaphragm *NF* resulted significantly affected by the factors *animal strain* and *time.* Diaphragm specimens hence presented a significant increase of the neuromuscular junction fatigability. For the same limitation explained above for the Soleus *IF*, the *NF* values for Soleus muscle were considered only until the 8th min of stimulation.

**Figure 7:**
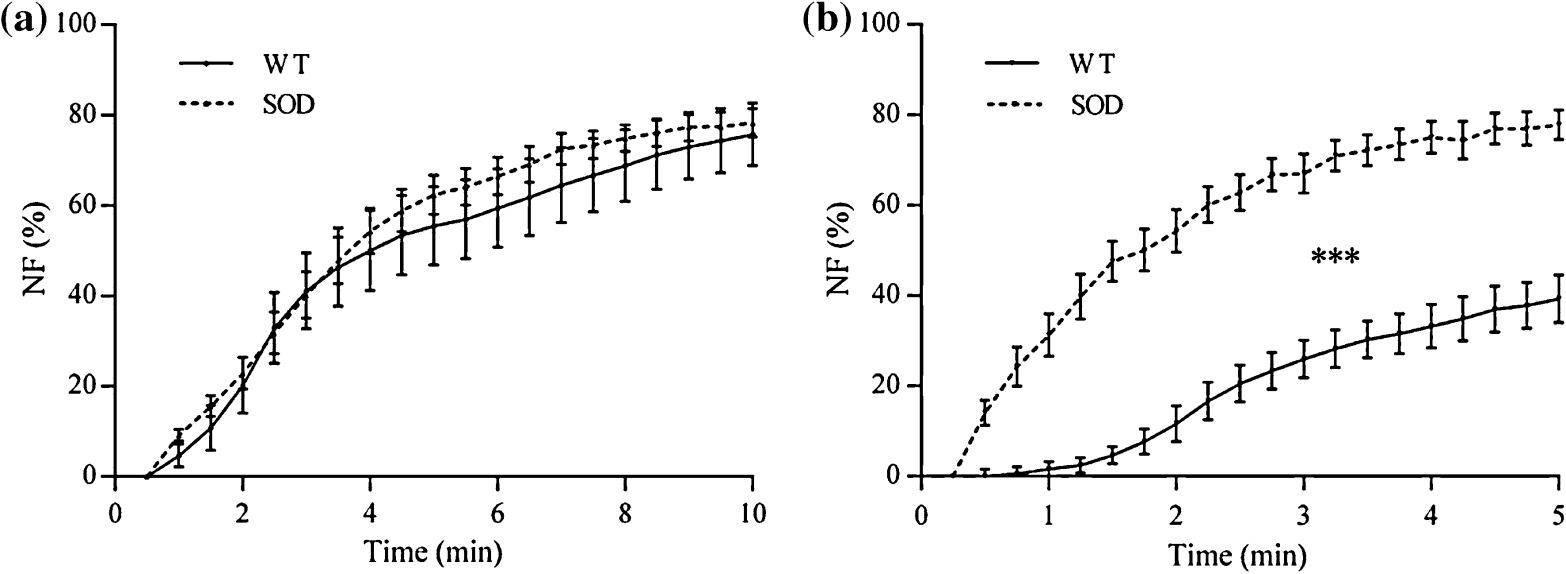
*Neurotransmission failure* computed at the firing frequency of 35 Hz for Soleus (a) and diaphragm (b). Values are mean ± SEM. SOD is for SOD1^G93A^. ***: *p* < 0.001 vs. WT

Similar results were reported for the *neurotransmission failure* measured at the firing frequency, as shown in Fig. 7. Once again Soleus *NF* (Fig. 7a) resulted affected only by the factor *time,* while diaphragm *NF* (Fig. 7b) was affected by both *animal strain* and *time* factors.

## Discussion

The aim of this work was to characterize the neuromuscular junction functionality of the SOD1^G93A^ transgenic mice,^21^ an animal model of ALS disease, at the very last stage of its lifespan. To do this, we tested two muscle types severely but differently affected by ALS, the Soleus and the diaphragm. The marked decrease we measured at this stage of the disease for the Soleus length and weight substantiates how much this muscle type is affected by the atrophic process induced by ALS. On the other hand, due to the arbitrariness with which they were cut, the diaphragm strips we tested cannot be considered representative of the muscle morphology, and it was not possible to correlate the anatomical measurements of the strips with the atrophic process on this muscle type. The absolute length and weight measured for the diaphragm strips are therefore meaningless, and only normalized parameters such as specific forces, *df*/*dt*, *IF* and *NF* can provide useful results.

Of note, the Soleus muscles in 130–140 day-old SOD1^G93A^ mice are almost completely paralyzed,^14^,^21^,^29^ while the diaphragm is functional until death. The results we obtained with direct stimulation experiments correlate well with these observations. Indeed, the Soleus muscle showed a significant decrease of the contractile kinetics and of the specific forces, while the diaphragm presented only modest alterations of the same properties.^24^ Despite this difference in the response to direct stimulation, both muscle types showed a significant defect in their contractile response when stimulated *via* the nerve. A slowing down of the contractile parameters, a dramatic reduction of the specific forces and an increased susceptibility to NMJ fatigue are, in fact, common features.

In addition to the dramatic decrease of specific forces, a change of the tetanic frequency occurred in both muscle types. Soleus and diaphragm tetanic frequency moved toward lower values, in agreement with the slowing down of the kinetic parameters measured with single pulse stimulations. Although the change of the tetanic frequency was limited for Soleus and diaphragm muscles when directly stimulated, it was quite big for diaphragm muscles when stimulated through the nerve. A pulse train frequency of 40 Hz was sufficient to elicit muscle maximum specific force. All together these results show that the two muscle types we tested have a slower phenotype than their WT counterparts.

Even though denervation is usually associated with a shift toward fast fibers,^9^ controversial results are also reported in the literature. For example, Agbulut *et al*.^1^ showed that sciatic nerve excision did not alter the WT Soleus MHC-1 protein content, but induced a slowing down of the functional kinetic parameters. A similar result was observed by Raffaello *et al*.^39^ in WT denervated EDL, who reported a slowing down of the kinetic parameters. Moreover, it has to be considered that the mouse model we studied in this work is a model of chronic denervation, where progressive death of motor neurons results both in denervation and in re-innervation of the muscles. In particular, it has been shown^20^,^38^ that, in SOD1^G93A^ mice, the large-diameter motor axons degenerate first, leading to denervation of fast muscle fibers. Selective degeneration of motor neurons with large-diameter axons was also shown by Hegedus *et al*.^22^,^23^, who reported early loss of fast fatigable motor units and survival of slow motor units. Therefore, the small-diameter motor axons which innervate slow muscle fibers are mainly responsible for re-innervation of vacant endplates, a process that can alter any fiber shift due to denervation. In view of all these reasons, we believe that the functional results here reported point out a comprehensive characterization of the muscle phenotype, thus involving fiber shift, denervation and re-innervation processes and fiber degeneration.

As regards to NMJ fatigability, it is well expressed by the *intratetanic fatigue*, which is an estimate of the muscle’s capability of maintaining force during a single tetanic contraction, and reflects the high-frequency fatigue. Our results showed that the stimulation through the nerve induced an increase of the susceptibility to fatigue up to 30% for Soleus and up to 50% for diaphragm muscles, respectively. This observation suggests that there might be some mechanism involved in the fast response of muscles to the stimuli, such as a decreased transmitter release. Of note, when subjected to direct stimulation, both WT and SOD1^G93A^ muscles did not undergo to any *IF*, supporting the hypothesis that a defect in biochemical signaling from the nerve occurred. Electrophysiological analysis performed by Mancuso *et al*. on SOD1^G93A^ mice plantar and tibialis anterior muscles demonstrated a slowing down of motor nerve fiber conduction,^32^ a result that supports our outcomes and confirms that common defects can be found in different muscle types.

As a matter of fact, the only parameter that exhibits different results for the two muscle types we tested is the *neurotransmission failure*, which is believed to play an important role in the development of fatigue, being related to axonal block of action potential propagation, decreased transmitter release or decreased end-plate excitability.^44^ The values of *NF* we measured with tetanic stimulation for WT diaphragms are in accordance with the ones from Personius and Sawyer.^36^ Since we measured *NF* at the end of the entire stimulation protocol, this outcome strengthens our hypothesis that the sequence of stimulation pulse trains and resting times proposed here did not induce fatigue in the tested muscles. *NF* measured at 35 Hz, on the contrary, differs from the values reported by Personius and Sawyer, who did not report alterations for WT diaphragms. Nonetheless, it has to be noted that they measured *NF* in a different way, testing the muscles with two separate paradigms, one stimulating directly the muscle and the other stimulating it through the nerve. Moreover, their results come from a low number of samples, thus showing high error bars. Indeed, other researchers^42^,^44^ reported for rat diaphragm values of *NF* that are in agreement with the ones we measured here.

Our experiments showed that *NF* remains unaltered in the Soleus and is significantly increased in the diaphragm, both at subtetanic and tetanic frequencies. This dissimilarity can be explained considering that at the disease end-stage the contractile capability is severely compromised in Soleus muscles, thus dominating any effects of NMJ deficits on the contractile measurements. In diaphragms, in contrast, muscle contractile properties are preserved even at the late stage of the pathology, and the defects due to neuromuscular transmission can therefore be distinguished. In fact, our results correlate well with biological, biochemical and electrophysiological evaluations already reported in the literature, according to which both muscle types should undergo *intratetanic fatigue*. A decrease of the estimated motor units number compared to the number of spinal cord motor neurons in gastrocnemius muscles,^35^ and a significant denervation of end-plates in transgenic Soleus, tibialis anterior, gastrocnemius and diaphragm muscles^8^,^19^,^46^ have been observed by others. In addition, it has been shown that, by day 80, the density of intact motor axons was markedly reduced in the ventral root, as well as a vacuolation of large motor neuron cell bodies, indicating that active degeneration was first observed.^19^,^46^ Finally, Llado *et al*.^28^ studied the electrophysiologic and pathologic properties of cervical motor neurons and phrenic nerves in mutant SOD1^G93A^ rats showing motor neuron loss, progressive reduction of phrenic nerve compound muscle action potential amplitudes, phrenic nerve fiber loss and diaphragm atrophy. Our findings confirmed the damages in the phrenic nerve fiber conduction. With our tests, we were able to discriminate between alterations in neuromuscular transmission and alterations in the diaphragm’s contractile capability, which remained substantially unaltered despite the compromised condition of SOD1^G93A^ animals.

In conclusion, we devised an experimental protocol to measure NMJ functionality in SOD1^G93A^ mice, and our results elucidated changes in NMJ functionality separately from those in the muscle contractile properties. The NMJ changes we measured with this technique could not clarify which part of the nerve stimulation pathway was altered, but the results clearly pointed out that a slowing down of the contractile kinetics, a decrease in the capability of generating force when stimulated through the nerve and an increase of *intratetanic fatigue* can be considered common signs of those muscle districts affected by ALS progression.
